# Predictors of 30-day and 90-day mortality among hemorrhagic and ischemic stroke patients in urban Uganda: a prospective hospital-based cohort study

**DOI:** 10.1186/s12872-020-01724-6

**Published:** 2020-10-08

**Authors:** Gertrude Namale, Onesmus Kamacooko, Anthony Makhoba, Timothy Mugabi, Maria Ndagire, Proscovia Ssanyu, John Bosco M. Ddamulira, Laetitia Yperzeele, Patrick Cras, Edward Ddumba, Janet Seeley, Robert Newton

**Affiliations:** 1 MRC/UVRI and LSHTM Uganda Research Unit, P.O Box 49, Entebbe, Uganda; 2 St. Francis Hospital Nsambya affiliated to Uganda Martyrs University, Kampala, Uganda; 3 School of Public Health, College of Health Sciences, Makerere University, Kampala, Uganda; 4 Stroke unit and Antwerp Neuro-Vascular Center, department of Neurology, University Hospital Antwerp, Antwerp Belgium and Research group on Translational Neurosciences, Faculty of Medicine and Health Sciences, University of Antwerp, Antwerp, Belgium; 5 Born Bunge Institute, University of Antwerp and Antwerp University Hospital, Department of Neurology, Antwerp, Belgium; 6 London School of Hygiene &Tropical Medicine, London, UK; 7 University of York, York, UK

**Keywords:** Hemorrhagic stroke, Ischemic stroke, Predictors, 30-day mortality, 90-day mortality, Glasgow coma scale (GCS), Modified ranking scale (mRS), National Institute of health stroke scale (NIHSS), Uganda

## Abstract

**Background:**

We report here on a prospective hospital-based cohort study that investigates predictors of 30-day and 90-day mortality and functional disability among Ugandan stroke patients.

**Methods:**

Between December 2016 and March 2019, we enrolled consecutive hemorrhagic stroke and ischemic stroke patients at St Francis Hospital Nsambya, Kampala, Uganda. The primary outcome measure was mortality at 30 and 90 days. The modified Ranking Scale wasused to assess the level of disability and mortality after stroke. Stroke severity at admission was assessed using the National Institute of Health Stroke Scale (NIHSS) and Glasgow Coma Scale (GCS). Examination included clinical neurological evaluation, laboratory tests and brain computed tomography (CT) scan. Kaplan-Meier curves and multivariate Cox proportional hazard model were used for unadjusted and adjusted analysis to predict mortality.

**Results:**

We enrolled 141 patients; 48 (34%) were male, mean age was 63.2 (+ 15.4) years old; 90 (64%) had ischemic and 51 (36%) had hemorrhagic stroke; 81 (57%) were elderly (≥ 60 years) patients. Overall mortality was 44 (31%); 31 (23%) patients died within the first 30 days post-stroke and, an additional 13 (14%) died within 90 days post-stroke. Mortality for hemorrhagic stroke was 19 (37.3%) and 25 (27.8%) for ischemic stroke. After adjusting for age and sex, a GCS score below < 9 (adjusted hazard ratio [aHR] =3.49, 95% CI: 1.39–8.75) was a significant predictor of 30-day mortality. GCS score < 9 (aHR =4.34 (95% CI: 1.85–10.2), stroke severity (NIHSS ≥21) (aHR = 2.63, 95% CI: (1.68–10.5) and haemorrhagic stroke type (aHR = 2.30, 95% CI: 1.13–4.66) were significant predictors of 90-day mortality. Shorter hospital stay of 7–13 days (aHR = 0.31, 95% CI: 0.11–0.93) and being married (aHR = 0.22 (95% CI: 0.06–0.84) had protective effects for 30 and 90-day mortality respectively.

**Conclusion:**

Mortality is high in the acute and sub-acute phase of stroke. Low levels of consciousness at admission, stroke severity, and hemorrhagic stroke were associated with increased higher mortality in this cohort of Ugandan stroke patients. Being married provided a protective effect for 90-day mortality. Given the high mortality during the acute phase, critically ill stroke patients would benefit from early interventions established as the post-stroke- standard of care in the country.

## Introduction

The trend towards an increasing burden of non-communicable chronic diseases (NCDs) including stroke in developing countries is of great concern [1]. The World Health Organization (WHO) estimates that by 2030, 80% of all stroke will occur in people living in low and middle income countries (LMICs), where it will account for 7.9% of all mortality [2, 3]. In sub-Saharan Africa (SSA), the burden of stroke is increasing as the population undergoes epidemiological and demographic change [4]. In Uganda, stroke is estimated to be one of the top five causes of adult deaths and accounts for 3.7% of all hospital admissions among adults [3]. All-stroke mortality from the available Ugandan hospital-based studies, is estimated to be between 30 and 40% at 1 month [5, 6], which is much higher than the 20% mortality reported in the rest of the world [7]. Hence, the increasing burden of stroke in the SSA region will put a huge burden on the already overstretched health care system and resources [8].

Stroke related mortality varies considerably between stroke types, regions and countries. For example in Uganda, 30-day mortality was found to be 43% compared to 27% in Gambia [6, 9]. While 90-day mortality was estimated at 50% in Tanzania compared to 29.4% in Korea [10, 11]. Furthermore, studies in Nigeria and South Africa, indicated that overall mortality at 1 month ranged between 30 and 35% [12, 13].. Reports have shown that mortality rates increased with increasing age and were higher for hemorrhagic stroke (HS) than for ischaemic stroke (IS) and, is often associated with a higher risk of early death [9, 14]. Previous studies have linked the excess mortality in patients with HS to more severe strokes [11, 15]. In Dublin, using the modified Ranking Scale (mRS), overall good outcome at 28 days was more common in IS (44.4%) compared with HS (26.8%) [16].

Predictors of stroke mortality have been identified in different studies [5, 9]. In Nigeria, 30-day mortality has been associated with advanced age, low level of consciousness on admission to hospital and higher National Institute of Health Stroke Scale (NIHSS) score [13]; presence of comorbid conditions such as diabetes mellitus (DM) has been associated with a higher long term mortality in Ghana [17]. In developing countries including Uganda, resources for stroke care and rehabilitation are still largely lacking particularly in lower health facilities [8]. However, the WHO has emphasized the importance of developing robust national surveillance systems to monitor stroke frequency and outcomes particularly in developing countries [18].

In SSA, few epidemiologic studies of stroke have explored and compared the outcomes of HS and IS separately. Most of the published evidence is cross-sectional with short-term follow-up [5, 6].

## Methods

### Aim, design and setting

We conducted a prospective hospital-based cohort study between December 2016 and March 2019 to investigate predictors of 30-day and 90-day mortality and functional disability among HS and IS patients at Nsambya Hospital, Kampala, Uganda. The hospital is a large urban tertiary referral hospital, which lies approximately 5 km southeast of Kampala, Uganda’s capital city. It receives patients from all parts of the country and offers medical care in all disciplines. Most of the patients pay personal private fees for treatment, while a minority has medical insurance covering the costs. Nsambya Hospital has around 19, 000 admissions per year and receives about 300 outpatients per day.

### Participants

The participants were patients who presented at Nsambya hospital- Kampala within 7 days of the onset of stroke symptoms, during the study period and, who met the WHO stroke definition: rapidly developing clinical signs of focal or global disturbance of cerebral function, lasting for more than 24 h or until death, with no apparent non-vascular cause [19] with neuro imaging confirmed stroke results.

### Eligibility criteria

The eligibility criteria for participation in the study included: 1) being an adult aged ≥18 years old; 2) confirmed cases of IS and HS on brain neuro-imaging; and 3) first-ever or recurrent stroke. We excluded participants who were: 1) unable to consent or for whom consent could not be obtained from a caregiver; (2) unable to communicate and without a caregiver respondent; (3) those who died within 24 h on the ward before neuroimaging was done; and 4) those who presented more than 7 days after onset of symptoms.

### Study procedures

All consecutive patients aged ≥18 years old with a confirmed diagnosis of HS or IS stroke were enrolled prospectively over a 2-year period. Both first-ever stroke and recurrent stroke patients were included. The study was based on the standardized WHO stepwise approach to stroke surveillance (STEPS) [20]. All information was collected by a trained medical team according to the STEPS Stroke Manual Instructions [20]. Within 24 h of admission, we obtained information about events that occurred within 7 days after the stroke event. Data on social demographic characteristics, social history, past medical history, stroke severity, pre-stroke functional ability, clinical characteristics and family history was collected. If patients were unable to respond to questions, family members served as proxy respondents. All participants underwent physical examination and assessment including recording blood pressure, physical function using a mRS [21] and neurological examination using the NIHSS [21] and Glasgow Coma Scale (GCS). Computed tomography (CT) scan was performed in all patients within the first 48 h after admission. Stroke types were differentiated based on CT scan interpretations into IS and HS.

### Follow up

All study participants were followed up prospectively at 30 days and 90 days by the study team. Participants were given an appointment to attend the neurology outpatient clinic post-stroke. Patients or their family members were contacted to remind them about the appointment date. Patients who failed to attend the follow-up visits were contacted and interviewed by telephone and, if not possible, their relatives were contacted, or a home visit was carried out. Information about functional status at 30 and 90 days using the mRS, stroke recurrence, mortality, mortality date and cause were recorded. Patients who had missed their day 30 or day 90 follow-up visits and did not present at the clinic within 30 days, and could not be contacted by phone, were considered as ‘lost-to-follow up’.

### Measurements

The primary outcome of the study was mortality at 30 and 90 days, and mortality was defined as the number of deaths among stroke patients which occurred within these time limits. The primary outcome was assessed using the mRS. For each patient, social demographic data (age, sex, level of education and marital status), information about presence of hypertension, DM, smoking habits, alcohol consumption, HIV infection, past medical history and a family history of Hypertension, DM and stroke were obtained. Hypertension was defined as either current use of antihypertensive medication or history of being diagnosed with hypertension prior to stroke or documented blood pressure of greater than or equal to 140 mmHg systolic or 90 mmHg diastolic. Diabetes mellitus was defined as current use of antidiabetic drugs, diagnose of type I or type II diabetes before stroke or a documented non-fasting blood glucose of greater than 11.1 mmol/L or fasting blood glucose of greater than 7.0 mmol/L outside of the acute phase of stroke (to exclude acute transient elevation of glucose as a stress response after stroke). Premorbid functional status was assessed using the mRS: (i) mRS 0–2 was considered good, (ii) mRS 3 was fair, and (iii) 4–5 indicated poor outcome. The length of hospital stay (LOS) for a single stroke hospitalization was defined as the time spent in hospital from admission until death or discharge to home. The severity of the neurological deficit at admission was assessed using the NIHSS. Higher figures correspond to higher stroke severity: (i) NIHSS 0–6 was considered mild; (ii) 7–12 was moderate; (iii) 13–20 was severe; (iv) ≥21 was very severe stroke. Level of consciousness was assessed using the GCS: (i) Good GCS (13–15); mild brain injury (alert), (ii) moderate GCS (9–12); moderate brain injury (drowsy), (iii) poor GCS (< 9); severe brain injury (unconscious).

### Sample size estimation

A sample size of 140 participants was needed to assess the primary outcome (30-day and 90-day mortality) of this study. The sample size was based on an anticipated 35% mortality for each of the two mortality measures [13]. An 80% power, 5% level of significance, 95% confidence interval were assumed. The sample was adjusted for a 10% rate of non-response. All eligible participants were consecutively enrolled during the study period.

### Data analysis

Data was double entered in OpenClinica (OC), cleaned, and exported to Stata15.0 (StataCorp, College Station, TX, USA) for analysis. We resolved discrepancies by checking the source documents for clarification on all possible confounders. Categorical demographic and clinical characteristics were summarized by counts and percentages. Continuous variables were summarized by means and standard deviations or medians and interquartile ranges. We used the Kaplan-Meier technique to estimate time to mortality after admission and presented the estimated mortality rates per 1000 person-days for the study variables. To compare the mortality distributions between the groups for the different variables of interest, a log rank test was used. Mortality rate was assessed within 30 days and at 90 days of follow-up. Cox proportional hazards regression was used to determine independent predictors of mortality among participants and these were expressed as estimated hazard ratios (HRs) with their corresponding 95% confidence intervals (CIs). In the bivariate proportional hazards analysis, variables significant at *p* ≤ 0.15 [22] were subsequently considered for the adjusted model. We considered age and gender as priori confounders; these were included in the adjusted model regardless of their unadjusted *p* values. The P- values represented the differences in the variable sub categories for 30- and 90-day mortality.

In adjusted modelling, variables whose *p*-value was less than 0.05 were considered independent predictors of stroke mortality. This was done both at 30 days and 90 days of follow-up. The proportional hazards assumption was tested using graphical inspection and tests for time trends of Schoenfeld residuals. In our model, we used a full model regression procedure to build the model and independent variables that were not significant at bivariate but interesting were added if adding them did not make the fit of the model significantly worse at the 5% level on a likelihood ratio test.

## Results

### Participants’ recruitment and outcome assessment profile

**Fig. 1:**
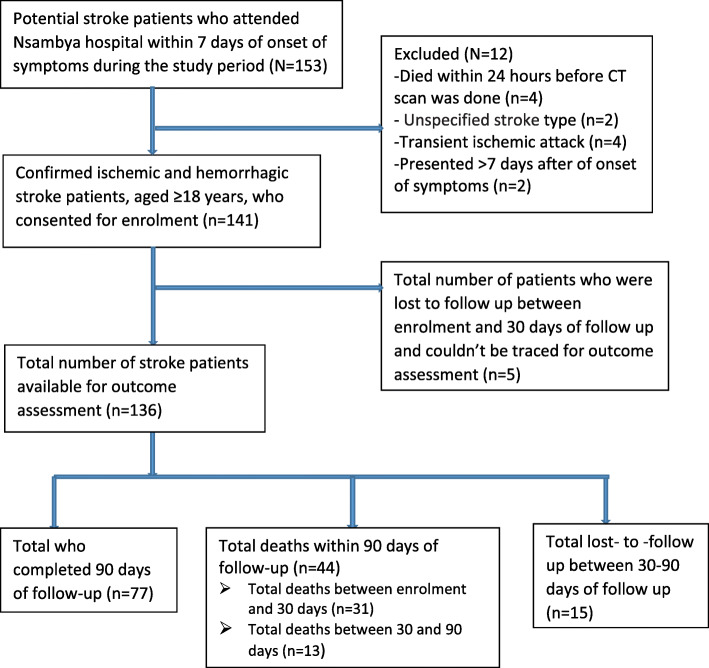
Summary of recruitment and outcome assessment profile for hemorrhagic and ischemic stroke patients in Kampala, Uganda

During the study period, 153 stroke patients attending Nsambya hospital were screened for eligibility. Of these, 12 (7%) were excluded: those who died within 24 h before CT scan was done (*n* = 4); those who had unspecified stroke (*n* = 2); those who had transient ischemic attacks (n = 4) and those who presented more than 7 days after onset of symptoms (n = 2). Thus a total of 141 patients with confirmed IS or HS that consented for enrolment were included, of these five were lost to follow up before outcome assessment at 30 days, 77 completed the 90 days of follow-up, a total of 44 patients died within 90 days post-stroke period (31 died between 0 and 30 days, while 13 died between > 30–90 days); 15 were lost to follow up between 30 and 90 days (Fig. 1).

### Participants’ baseline characteristics

**Fig. 2:**
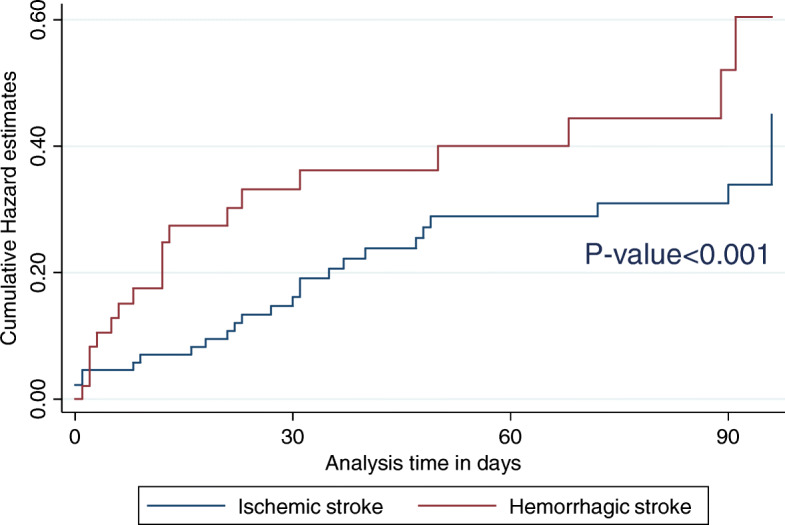
Cumulative stroke mortality among hemorrhagic and ischemic stroke patients at 90 days of follow up in Kampala, Uganda

Of the 141 participants included in the analysis, 48 (34%) were male, mean age was 63.2 (+ 15.4) years old, 90 (64%) had IS, while 51 (36%) had HS; 65 (46%) attained secondary education and above; 81 (57%) were ≥ 60 years. The mean length of hospital stay was 9.4 (SD ± 10.7) days. Median time between the onset of symptoms and medical attention for IS patients was higher (2 days (Interquartile range [IQR]: 1–4 days) compared to HS (1 day [IQR]: 1–5 days) (Table 1). Overall mortality was 44 (31%); 31 (23%) patients died within the first 30 days post-stroke and, an additional 13 (14%) died within 90 days post-stroke. The mean follow-up duration was 61 (SD ± 34) days, with a total of 8274 person days of follow up at 90 days. The overall mortality for HS was 19 (37.3%) and 25 (27.8%) for IS (Table 2). Figure 2 shows cumulative mortalities during the 3 months follow-up period according to stroke type. We observed a statistically significant higher mortality rates among females compared to males at 30 days (*p* = 0.039). Thirty-day and 90-day mortality increased with increasing age at diagnosis (*p* = 0.036). In unadjusted analyses, individuals aged ≥60 years old, females, patients without formal education, patients who were divorced or separated, severe stroke NIHSS > 21, longer hospital stay > 14–29 days, low GCS < 9 and HS type presented higher mortality rates across 90-day follow up period compared to other subgroups (Table 1).

**Table 1:** Mortality rates according to socio demographic and clinical characteristics of patients with ischemic and haemorrhagic stroke during 30-day and 90-day follow-up in Kampala, Uganda

Baseline characteristics	Overall mortality ***N*** = 141 n (col %)	30-day mortality ***N*** = 136 (n (%))	30-day mortality rate (95% CI) (Deaths/1000 person days)	***P***-value	90-day mortality ***N*** = 90 (n (%))	90-day mortality rate (95% CI) (Deaths/1000 person days)	P-Value
**SOCIO DEMOGRAPHIC CHARACTERISTICS**							
**Age (years)**				0.036			0.039
- → < 60	60 (43)	8 (5.8)	4.4 (2.21–8.83)		5 (5.6)	3.4 (1.96–5.82)	
- → ≥ 60	81 (57)	23 (16.9)	10.4 (6.93–15.7)		8 (8.9)	7.0 (4.92–9.96)	
**Gender**				0.039			0.101
- → Male	48 (34)	7 (5.1)	3.9 (1.77–8.77)		5 (5.6)	3.6 (1.98–6.46)	
- → Female	93 (66)	24 (17.6)	10.0 (6.78–14.8)		8 (8.9)	6.3 (4.51–8.92)	
**Marital status**				0.205			0.037
-Married	67 (48)	11 (8)	5.6 (3.08–10.0)		4 (4.4)	3.6 (2.15–5.92)	
-Widowed	49 (35)	13 (9.5)	9.9 (5.76–17.1)		2 (2.2)	5.5 (3.34–9.18)	
Divorced/separated	16 (11)	5 (3.7)	13.9 (6.24–30.9)		5 (5.5)	11.6 (6.22–21.5)	
-Never married	9 (6)	2 (1.4)	3.42 (0.48–24.3)		2 (2.2)	8.1 (3.03–21.5)	
**Education level**				0.396			0.823
-No education	20 (14)	6 (4.4)	10.9 (4.91–24.3)		–	5.3 (2.38–11.8)	
-Primary	56 (40)	8 (5.9)	5.5 (2.87–10.6)		8 (8.8)	4.7 (2.88–11.8)	
-Secondary +	65 (46)	17 (12.5)	8.7 (5.34–14.2)		5 (5.5)	5.9 (3.87–8.93)	
**Smoking**				0.020			0.125
- → No	31 (22)	2 (1.5)	2.0 (0.50–8.05)		4 (4.4)	3.1 (1.40–6.92)	
- → Yes	110 (78)	29 (21.3)	9.6 (6.67–13.8)		9 (10.0)	6.0 (4.36–8.23)	
**Alcohol use**				0.071			0.057
-Low risk drinkers	105 (74)	27 (19.9)	9.3 (6.37–13.5)		10 (11.1)	6.4 (4.64–8.83)	
-Harmful/high risk	36 (26)	4 (2.9)	3.6 (1.36–9.60)		3 (3.3)	2.8 (1.34–5.89)	
**Family history of DM**				0.634			0.901
- → Yes	103 (73)	17 (12.5)	7.3 (4.71–11.2)		9 (10.0)	5.3 (3.70–7.48)	
- → No	38 (27)	14 (10.3)	8.7 (4.70–16.2)		4 (4.4)	5.5 (3.17–9.40)	
**Family history of HTN**				0.906			0.557
- → Yes	92 (65)	21 (15.4)	7.9 (5.20–12.2)		11 (12.2)	6.0 (4.26–8.52)	
- → No	33 (23)	7 (5.1)	7.9 (3.77–16.6)		1 (1.1)	4.3 (2.12–8.49)	
- → Don’t know	16 (12)	3 (2.2)	6.0 (1.94–18.6)		1 (1.1)	3.7 (1.39–9.90)	
**CLINICAL CHARACTERISTICS**							
**Self-reported history of HTN**				0.995			0.563
- → Yes	83 (59)	18 (13.2)	7.7 (4.92–12.1)		8 (8.8)	5.0 (3.39–7.42)	
- → No	58 (41)	13 (9.6)	7.7 (4.40–13.6)		5 (5.5)	5.8 (3.69–9.07)	
**Self-reported history of DM**				0.489			0.333
- → Yes	28 (20)	7 (5.1)	9.6 (4.78–19.1)		4 (4.4)	7.2 (4.08–12.6)	
- → No	113 (80)	24 (17.6)	7.2 (4.81–10.9)		9 (10)	4.9 (3.43–6.85)	
**BP ≥ 140/90**				0.292			0.272
- → Yes	82 (58)	21 (15.4)	8.9 (5.86–13.9)		9 (10.0)	6.1 (4.26–8.71)	
- → No	59 (42)	10 (7.4)	6.0 (3.20–11.1)		4 (4.4)	4.2 (2.48–7.06)	
**Obese**				0.766			0.372
- → Yes	29 (21)	6 (4.4)	6.9 (3.09–15.3)		1 (1.1)	4.0 (1.90–8.36)	
- → No	112 (79)	25 (18.4)	7.9 (5.37–11.8)		12 (13.3)	5.7 (4.11–7.84)	
**HIV infection**				0.294			0.751
- → Yes	27 (19)	2 (1.5)	4.8 (1.79–12.7)		4 (4.4)	4.9 (2.46–9.84)	
- → No	114 (81)	27 (20.5)	8.5 (5.83–12.4)		9 (10.0)	5.4 (3.91–7.51)	
**Duration of hospitalization**				0.015			0.004
Mean duration ±SD	9.4 (10.7)						
- < 7 days	78 (55)	17 (12.5)	7.4 (4.54–12.1)		5 (5.5)	4.4 (2.89–6.80)	
-7–13 days	37 (26)	6 (4.4)	4.3 (1.78–10.2)		5 (5.5)	4.7 (2.58–8.40)	
-14–29 days	17 (12)	7 (5.2)	20.2 (10.5–38.8)		3 (3.3)	17.5 (9.7–31.6)	
- ≥ 30 days	9 (7)	1 (0.5)	4.2 (0.59–30.0)		–	1.8 (0.26–13.0)	
**Glasgow Coma Scale (GCS)**				< 0.001			< 0.001
Mean GCS ± SD	12 (2.8)		11.0 (3.2)			13.3 (2.4)	
- < 9	21 (15)	11 (8.1)	28.6 (15.8–51.6)		2 (2.2)	20.9 (12.1–35.9)	
- ≥ 9	120 (85)	20 (14.7)	5.5 (3.55–8.54)		11 (12.2)	4.1 (2.85–5.76)	
**Stroke severity at enrolment**							< 0.001
Mean NIHSS ±SD	15 (10.1)		20.0 (10.7)	< 0.001		13.2 (9.0)	
-NIHSS 0–12	66 (47)	7 (5.1)	2.8 (1.28–6.32)		4 (4.4)	2.4 (1.33–4.35)	
-NIHSS 13–20	33 (23)	6 (4.4)	7.8 (3.74–16.4)		4 (4.4)	5.3 (2.87–9.91)	
-NIHSS ≥21	42 (30)	18 (13.2)	17.9 (11.3–28.3)		5 (5.5)	12.6 (8.34–18.9)	
**Stroke type**				0.079			0.123
Ischemic stroke	90 (64)	16 (11.8)	5.92 (3.63–9.67)		9 (10.0)	4.4 (2.98–6.53)	
Hemorrhagic stroke	51 (36)	15 (11.0)	11.4 (6.89–18.9)		4 (4.4)	7.3 (4.65–11.4)	
**History of stroke**				0.334			0.977
-Yes	20 (14)	6 (4.4)	11.3 (5.08–25.2)		–	5.4 (3.90–7.37)	
-No	121 (86)	25 (18.4)	7.2 (4.85–10.6)		13 (14.4)	5.1 (2.28–11.3)	
**Pre-stroke functional status**				0.998			0.804
-mRS 0–2	132 (93)	29 (21.3)	7.7 (5.37–11.1)		12 (13.3)	5.3 (3.92–7.23)	
-mRS 3	5 (4)	1 (0.7)	7.4 (1.04–52.2)		1 (1.1)	7.5 (1.87–29.0)	
-mRS 4–5	4 (3)	1 (0.7)	7.9 (1.11–55.9)		–	3.3 (0.46–23.1)	
**Functional status at enrolment**				0.016			0.009
-mRS 0–3	31 (22)	2 (1.5)	1.9 (0.48–7.69)		2 (2.2)	1.8 (0.66–4.68)	
-mRS 4–5	110 (78)	29 (21.3)	9.8 (6.77–14.0)		11 (12.2)	6.7 (4.89–9.09)

**Table 2:** Cumulative mortality rates at 30 and 90-days according to stroke type among ischemic and haemorrhagic stroke patients in Kampala, Uganda

Mortality Rates/1000 Person days			
Characteristic	Person time in days	Mortality n (%)	Mortality rate (95% CI)
**Timing**			
Overall (at 90 days) (n = 141)	8274.1	44 (31)	5.32 (3.96–7.15)
0–30 days (n = 136)	4015.1	31 (23)	7.72 (5.43–10.98)
31–90 days (n = 90)	4259.0	13 (14)	3.05 (1.39–4.71)
**Stroke type**			
Hemorrhagic stroke (*n* = 51)	2606.0	19 (37.3)	7.29 (4.65–11.43)
Ischemic stroke (n = 90)	5668.1	25 (27.8)	4.41 (2.98–6.53)

### Functional status and stroke severity

The majority 132(93%) of patients were fully independent (mRS 0–2) before stroke. On admission, mean NIHSS was 15 (SD ±10.1) and 42(30%) of the patients had severe stroke (NIHSS ≥21). The mean GCS was 12 (SD ±2.8), and only 21 (15%) had GCS < 9 in all stroke, majority with a GCS < 9 died within 1 month; 58 (41%) had severe disability (mRS 4–5) at 30 days post-stroke and 38 (36%) at 90 days (Table 1).

### Predictors of 30-day and 90-day mortality

Univariate analysis showed that age ≥ 60 years (uHR = 2.3, 95% CI: 1.03–5.16), smoking (uHR = 4.64, 95% CI: 1.11–19.4), stroke severity (NIHSS > 21) (uHR = 5.9, 95% CI: 2.33–14.9), prolonged hospital stay of ≥14 days (uHR = 2.64, 95% CI: 1.17–6.00), lower GCS < 9 (uHR = 4.71, 95% CI: 2.24–9.87), and poor functional status at enrolment (mRS 4–5) (uHR = 4.89, 95% CI: 1.17–20.5), significantly increased the risk of mortality at 30 days. Age ≥ 60 years old (uHR = 1.95 (95% CI: 1.02–3.73), longer hospital stay of 14–29 days (uHR = 3.2 (95% CI: 1.53–6.78), lower GCS < 9 (uHR = 4.33, 95% CI: 2.24–8.36), stroke severity (NIHSS ≥21) (uHR = 4.66 (95% CI: 2.26–9.58) and poor functional status at enrolment (mRS 4–5) (uHR = 3.58, 95% CI: 1.28–10.0) increased the risk of mortality at 90 days (Table 3).

**Table 3:** Predictors of mortality in patients with ischemic and haemorrhagic stroke during a 30-day and 90-day follow up in Kampala, Uganda

	30 -day mortality			90 -day mortality		
Variable	uHR (95% CI)	LRT p-value	aHR (95%CI)	uHR (95% CI)	LRT p-value	aHR (95%CI)
**Age (years)**		0.032			0.044	
< 60	1		1			1
≥ 60	**2.30 (1.03–5.14)**		1.11 (0.45–2.75)	**1.95 (1.02–3.73)**	0.092	0.92 (0.41–2.06)
Gender, male	0.41 (0.17–0.98)	0.031	0.54 (0.20–1.46)	0.57 (0.29–1.13)		0.78 (0.32–1.90)
**Marital status**		0.222			0.063	
-Never married	1			1		1
-Widowed	2.79 (0.36–21.3)			0.71 (0.24–2.14)		0.26 (0.07–1.01)
-Divorced/separated	3.89 (0.47–32.2)			1.42 (0.44–4.54)		0.32 (0.07–1.43)
-Married	1.59 (0.21–12.3)			0.46 (0.15–1.40)		**0.22 (0.06–0.84)**
**Education level**		0.395			0.824	
-No education	1			1		
-Primary	0.52 (0.18–1.46)			0.92 (0.36–2.34)		
-Secondary & above	0.81 (0.32–2.07)			1.12 (0.45–2.76)		
**Smoking**		0.009			0.105	
No	1		1	1		1
Yes	**4.64 (1.11–19.4)**		3.85 (0.88–16.8)	1.96 (0.82–4.59)		1.68 (0.69–4.11)
**BP ≥ 140/90**		0.288			0.267	
- No	1			1		
- Yes	1.49 (0.70–3.17)			1.42 (0.75–2.69)		
**Obese**		0.764			0.357	
- No	1			1		
- Yes	0.87 (0.36–2.13)			0.69 (0.31–1.56)		
**-HIV infection**		0.270			0.749	
- No	1			1		
- Yes	0.58 (0.20–1.64)			0.88 (0.41–1.90)		
**-Length of hospital stay**		0.040			0.017	
- < 7 days	1		1	1		1
-7–13 days	0.58 (0.21–1.60)		**0.31 (0.11–0.93)**	1.03 (0.50–2.15)		0.55 (0.24–1.26)
-14–29 days	**2.64 (1.17–6.00)**		1.02 (0.37–2.81)	**3.22 (1.53–6.78)**		1.10 (0.42–2.82)
- ≥ 30 days	0.58 (0.08–4.36)		0.20 (0.02–1.71)	0.43 (0.06–3.18)		0.13 (0.02–1.06)
**Glasgow Coma Scale**		< 0.001			< 0.001	
- ≥ 9	1		1	1		1
**- < 9**	**4.71 (2.24–9.87)**		**3.49 (1.39–8.75)**	**4.33 (2.24–8.36)**		**4.34 (1.85–10.2)**
**Stroke severity at enrolment**		< 0.001			< 0.001	
-NIHSS 0–12	1		1	1		1
-NIHSS 13–20	2.71 (0.91–8.05)		1.48 (0.45–4.91)	2.24 (0.95–5.27)		1.85 (0.68–5.03)
-NIHSS ≥21	**5.89 (2.33–14.8)**		2.73 (0.85–8.80)	**4.66 (2.26–9.58)**		**2.63 (1.68–10.5)**
**Pre-stroke functional status**		0.998			0.798	
-mRS 0–2^a^	1		1	1		1
-mRS 3	0.95 (0.13–7.00)		0.29 (0.03–2.84)	1.35 (0.33–5.60)		0.36 (0.07–1.79)
-mRS 4–5	1.03 (0.14–7.59)		0.37 (0.03–4.51)	0.61 (0.08–4.46)		0.40 (0.04–3.71)
**Stroke type**		0.089			0.133	
Ischemic stroke^a^	1		1	1		1
Haemorrhagic stroke	1.86 (0.92–3.76)		1.95 (0.90–4.25)	1.59 (0.88–2.89)		**2.30 (1.13–4.66)**
**Functional status at enrolment**		0.006			0.004	
-mRS 0–3^a^	1					
-mRS 4–5	**4.89 (1.17–20.5)**		2.55 (0.52–12.6)	**3.58 (1.28–10.0)**		2.15 (0.66–7.08)

^a^ reference category; *uHR* unadjusted hazard ratios, *aOR* adjusted hazard ratios, *CI* Confidence interval, *BP* Blood pressure, *LRT* likelihood ratio test, *NIHSS* National Institute of Health Stroke Scale, *mRS* Modified Rankin Scale

After adjustment for age and sex in a multivariable analysis, lower GCS < 9 (aHR =3.49 (95% CI: 1.39–8.75) was a significant predictor of 30-day mortality, whereas lower GCS of < 9 (aHR =4.34 (95% CI: 1.85–10.2), stroke severity (NIHSS ≥21) (aHR) = 2.63 (95% CI: (1.68–10.5) and haemorrhagic stroke type (aHR = 2.30, 95% CI: 1.13–4.66) were significant predictors of 90-day mortality. Shorter hospital stay (< 14 days) (aHR = 0.31 (95% CI: 0.11–0.93) and being married (aHR = 0.22 (95% CI: 0.06–0.84) were associated with reduced 30- and 90-day mortality respectively (Table 3).

## Discussion

In this study at a referral hospital in urban Uganda, the overall short and medium term mortality associated with stroke was relatively high compared to other settings [23, 24] especially in the acute phase, and the functional status of patients after stroke was poor. These results are in accordance with findings from other studies in SSA [5, 25], and confirm the trends reported by the global burden of disease (GBD) study in 2013, regarding the burden of stroke in SSA [7]. This suggests that there is still much to be done in the prevention and management of stroke, including the provision of specialized stroke care for early competent care, as well as the establishment of well-designed rehabilitation centres within the SSA region.

The relatively high mortality rate in this study is comparable to other hospital based studies in SSA [9, 14] and is substantially higher than observed in high-income countries [23, 24]. The dissimilarities may be explained by the differences in stroke care and management as well as type of rehabilitation programs between countries. However in Uganda, the presence of continuum of poverty [26] may lead to poor accessibility to hospital care, late presentation of severe stroke and poor compliance to medication with eventual poor health outcomes [8]. A recent study in Uganda found that only 27% of medicines and 32% of diagnostic tests for diabetes and cardiovascular disease were affordable by most people [27]. While poverty remains a long-term problem in health care, simple interventions such as education of patients and their caregivers in recognising early symptoms and improving referral systems can improve stroke outcomes.

In addition to the high mortality, we also observed severe disabilities among our patients during follow up. The lack of stroke rehabilitation facilities, the inadequate home-based care support for stroke patients and the high levels of poverty within the communities [8, 28] may also have contributed towards the poor outcomes among our stroke patients. In this cohort, sometimes a patient would be withdrawn from the hospital against medical advice due to fear of the high medical bills [28]. Moreover, the majority of patients who participated in this study catered for their own medical bills and only a few had health insurance. A recent qualitative study [28] conducted in Uganda, found that some patients were taken to churches or traditional healers for treatment where they would eventually die or lost to follow up. The concern is that, poor stroke outcomes may increase in the region if interventions for prevention and post-stroke care are not put in place.

The study also shows that mortality was particularly high in the first 30 days, which is comparable to other studies in SSA [5, 25]. Previous international research has shown that after stroke, nearly two-thirds of patients develop at least one complication [10, 29]. The hematoma expansions, oedema formation, and intra-ventricular haemorrhage leading to increased intracranial pressure are likely contributors to the acute mortality [30]. These complications cannot be managed at home or many of the primary care facilities available across SSA. This suggests that interventions focusing on prevention could potentially have the highest impact.

We also showed that lower GCS < 9 was a significant predictor for both 30 and 90-day mortality and, that stroke severity (NIHSS ≥21) and HS were significant predictors of 90-day mortality. Similar correlations between GCS < 9, high NIHSS score (≥21), HS and mortality rates have been previously reported [13, 31]. In this study, most of our participants were quite ill with mean NIHSS significantly high and low levels of consciousness (GCS < 9) on admission. In Africa, factors including long distances to health care and poor accessibility to transport are likely to lead to greater delay among patients reaching hospital than in developed countries [8, 24]. The late presentation to care for our patients with severe strokes has been reported to influence high mortality particularly during the acute phase [32]. The development of healthcare approaches that can allow early access to specialized neurological care, availability of rehabilitation, effective treatment, and improved knowledge on factors associated with a higher risk of death should contribute to decreasing the considerable burden of stroke in SSA. However, in this cohort, although stroke patients had no access to formal rehabilitation services in their communities, family members were often dedicated in their provision of care to their loved ones at home [28].

Consistent with previous findings [33], our study showed that marriage provided a protective effect for mortality at day 90. In developing countries like Uganda, marriage can be viewed as a fundamental culture for caring and supporting stroke survivors in the community [34]. Some researchers suggest that this is partly because spouses can provide some stable financial, social, and psychological support after stroke [35]. Evidence from one study [36] indicates that stroke survivors who received high amounts of emotional support from their spouses experienced superior functional recovery.

Mortality was higher in patients with HS compared to patients with IS, confirming existing reports in the SSA population [37]. Reports have shown that HS is related to a higher risk of acute complications such as hematoma expansion and intracranial hypertension than in patients with IS, thus leading to severe strokes in these patients [38]. The high proportion of HS deaths may also be largely explained by the high prevalence of hypertension in the country along with the low awareness and poor compliance to antihypertensive therapies [39]. This finding reflects the need for low-cost treatments to be made widely available in the country, sufficient coverage of emergency services, surgical interventions, improvement of the national referral system, and intensive nationwide sensitization on stroke.

Our study had some limitations. First, this was a single site hospital-based study in an urban setting, with a short duration of recruitment and follow-up up to 90 days. Therefore, the findings may not reflect the overall picture of mortality in the population. Patients with very severe strokes may have died before reaching the hospital, whilst mild self-limiting cases may never have presented for admission. Further population-based prospective studies are warranted to evaluate longer term stroke mortality including both urban and rural settings. Second, an inclusion bias could have occurred attributable to the urban hospital-based design of the study. The majority of the patients came from an urban population who could afford hospitalization. Mortality rates may differ from a rural or lower income population. Third, some patients were lost to follow-up, and no outcome could be ascertained, which may have led to underestimates of mortality. Fourth, some of the predictor variable analyses were hampered by the small sample size, with few cases of hemorrhagic stroke, which made it difficult to identify possibly relevant predictors. Fifth, blood pressure measurements were performed upon admission, and should ideally have been repeated outside of the acute window to exclude temporary ischemia induced hypertension. Finally, there was a lack of subject awareness of some stroke risk factors, and we depended on the next of kin’s report for patients with severe strokes.

### Policy implications

Measures for secondary prevention of stroke, particularly proper treatment, referral, and intensive follow-up including drug compliance are desperately needed in Africa. We hope that taking into account the predictors for mortality for each stroke type in the present study, individual patient management can be better optimized and outcomes improved in Uganda. Further studies to examine the relationship between the type of stroke and immediate cause of death are warranted. The knowledge of the immediate cause of death and other contributory factors will greatly enhance effective management of acute stroke.

## Conclusions

Mortality is high during the acute phase of stroke. Low levels of consciousness, stroke severity, longer hospital stay, and hemorrhagic stroke type were associated with higher mortality in this cohort of Ugandan stroke patients. Being married was associated with lower 90-day mortality. Given the high mortality during the acute phase, critically ill stroke patients would benefit from early interventions established as the post-stroke- standard of care in the country.

## Data Availability

The data used to support the findings of this study are available at MRC/UVRI and LSHTM Uganda Research Unit, and are available from the corresponding author upon reasonable request and with permission from MRC/UVRI and LSHTM Uganda Research Unit.

## Abbreviations

BP: Blood pressure
CI: Confidence interval
CT: Computed tomography
DM: Diabetes mellitus
GCS: Glasgow coma scale
HIV: Human immunodeficiency virus
HTN: Hypertension
HS: Hemorrhagic stroke
IQR: Interquartile range
IS: Ischemic stroke
LMICs: Low and middle income countries
LSHTM: London school of hygiene and tropical medicine
mmHg: Millimetres of mercury
MRI: Magnetic resonance imaging
MRC: Medical Research Council
mRS: Modified Rankin scale
NCDs: Non-communicable diseases
NIHSS: National Institute of health stroke scale
HR: Hazard ratio
SD: Standard deviation
SSA: Sub-Saharan Africa
STEPs: Stepwise approach to chronic disease risk factor surveillance
UNCST: Uganda National Council of science technology
UVRI: Uganda virus research institute
WHO: World Health Organisation
